# Why Machiavellianism Matters in Childhood: The Relationship Between Children's Machiavellian Traits and Their Peer Interactions in a Natural Setting

**DOI:** 10.5964/ejop.v11i3.957

**Published:** 2015-08-20

**Authors:** Loren Abell, Pamela Qualter, Gayle Brewer, Alexandra Barlow, Maria Stylianou, Peter Henzi, Louise Barrett

**Affiliations:** a School of Psychology, University of Central Lancashire, Preston, United Kingdom; b School of Education, University of Manchester, Manchester, United Kingdom; c School of Psychology, Neapolis University, Pafos, Cyprus; d Psychology Department, University of Lethbridge, Lethbridge, Canada; Aalborg University, Aalborg, Denmark; College of New Rochelle, New York, USA

**Keywords:** Machiavellianism, peer relations, observation, aggression, peer rejection

## Abstract

The current study investigated the association between Machiavellianism and children’s peer interactions in the playground using observational methods. Primary school children (N = 34; 17 female), aged 9 to 11 years, completed the Kiddie Mach scale and were observed in natural play during 39 recesses (average observed time = 11.70 hours) over a full school year. Correlations for boys revealed that Machiavellianism was related to more time engaging in direct and indirect aggression, being accepted into other peer groups, and accepting peers into their own social group. Correlations revealed that for girls, Machiavellianism was associated with lower levels of indirect aggression, less time being accepted into other groups and less time accepting and rejecting other children into their own group. This preliminary pilot study indicates that Machiavellianism is associated with children’s observed social behaviour and aims to promote future observational research in this area.

## Introduction

Few studies have considered the development of Machiavellianism or the role of Machiavellianism in children’s social interactions. Existing questionnaire based studies indicate that children high on Machiavellianism are more likely to engage in indirect aggression, but these studies do not examine children’s normative behaviour with peers. Our preliminary study addresses this limitation by investigating the association between Machiavellianism and children’s actual behaviour in a naturalistic playground environment across a full school year.

### Machiavellianism

Machiavellianism is characterised by cynical beliefs, a manipulative interpersonal style, and emotional detachment (Christie & Geis, 1970). Those with high levels of Machiavellianism are low in conscientiousness, agreeableness, trait emotional intelligence^i^ and empathy (Austin, Farrelly, Black, & Moore, 2007; Wai & Tiliopoulos, 2012) and demonstrate a willingness to exploit other people in order to attain individual goals (Wilson, Near, & Miller, 1996). Machiavellian adults employ a range of tactics in order to manipulate others (Jonason & Webster, 2012), including making their target feel ashamed, embarrassed, or guilty (Austin et al., 2007). Being high on Machiavellianism makes it more difficult to interact with others in a positive way and form and maintain high quality friendships (Lyons & Aitken, 2010).

#### Machiavellianism among Children

Relatively few studies have investigated the behaviour of children high on Machiavellianism or the social implications of Machiavellianism in childhood. Experimental studies have demonstrated that Machiavellianism is associated with children’s manipulative interpersonal behaviour and deception (Braginsky, 1970; Nachamie, 1969, as cited in Christie & Geis, 1970). Self-report studies incorporating peer or teacher reports also indicate that Machiavellianism is associated with specific social behaviour, such that children scoring higher on Machiavellianism are reported to be less prosocial and more aggressive towards peers, and are more likely to be categorised as bullies (Slaughter & Pritchard, 2000, as cited in Repacholi, Slaughter, Pritchard, & Gibbs, 2003; Sutton & Keogh, 2000).

However, peer ratings of Machiavellian children are inconsistent, with researchers reporting that Machiavellian children are both popular (Hawley, 2003) and less well liked (Palmen, 2009) by peers. Such inconsistencies may be partly explained by the use of both prosocial and coercive strategies by Machiavellian children (Hawley, 2003) that extend to the use of cooperative and competitive strategies in adulthood (Wilson et al., 1996). Teacher ratings relate Machiavellianism to children’s indirect aggression (Kerig & Stellwagen, 2010), reflecting the preference for indirect manipulation observed in adult samples (Austin et al., 2007).

Those studies are suggestive of an association between Machiavellianism and children’s peer relationships, but are limited by the reliance on self-report and other-report measures. Such methods require retrospective reporting and may reflect social bias (Child & Nind, 2013; Ostrov & Keating, 2004). To overcome these limitations, in the current study, we directly observed children in a naturalistic playground environment, which provides important opportunities to observe the role of Machiavellianism in peer interaction.

#### The Current Study

The present study expands previous questionnaire-based research by focusing on Machiavellianism and children’s directly observed normative behaviour over the course of a year. By using an observational design we start to develop a picture of how Machiavellianism is associated with *actual* behaviour and can move away from relying on (self and other) reports of behaviour. We further considered whether Machiavellianism was associated with gender differences in children’s behaviour based on previous observational studies that identified gender differences in interaction stability and frequency (Martin & Fabes, 2001) and characteristics of play (Pellegrini, Blatchford, Kato, & Baines, 2004). Based on previous studies that examined self-reports and teacher-reports of Machiavellianism and behaviour (Kerig & Stellwagen, 2010; Sutton & Keogh, 2000), we hypothesized the following: (1) Machiavellianism will be associated with the amount of indirect aggression (Kerig & Stellwagen, 2010) which is more prevalent among girls (Archer, 2004); we expect higher Machiavellianism scores to be associated with spending more time engaging in indirect aggression, with a stronger relationship for girls; and (2) Machiavellianism will be associated with actual amount of social rejection from others in the playground, with a stronger relationship for girls based on the predicted use of indirect aggression.

In addition, we investigated the relationship between Machiavellianism and social monitoring. The literature suggests Machiavellian adults engage in protective self-monitoring (Rauthmann, 2011), but little is known about the role of monitoring others. This may be particularly important to children in playground settings that involve varied social engagement, from competition and conflict to prosocial behaviour and cooperation. Such monitoring may influence a child’s decision to interact. In this study, we investigated social monitoring of peers outside the target child’s group. Machiavellianism is characterised by suspicion of others (Christie & Geis, 1970), and, in children, is associated with anxiety which originates from increased vigilance for opportunities to engage in manipulation (Poderico, 1987). So, we expected Machiavellianism to be associated with monitoring of those outside the immediate group because non-group members may be viewed as a threat to the child’s social position; monitoring may reduce the likelihood of being a target of manipulation or exploitation by another child. We expect girls to engage in this behaviour more as monitoring of others may facilitate the use of indirect aggression.

## Method

### Participants

Thirty four children (17 boys, 17 girls) aged 9 to 11 years from The Lancashire Longitudinal Study of Social and Emotional Development participated. Mean ages at the start and end of the study were 9 years-10 months (*SD* = 5 months) and 10 years-6 months (*SD* = 5 months) respectively. Children came from six schools, representative of those across the UK according to the government Index of Multiple Deprivation. Participation was secured by active parental consent. Children who did not take part in the study were often observed in interaction with children in the study. The parents of such children were informed that their child’s behaviour would be recorded, but not coded. All parents were told that the recordings would be destroyed at the end of the study. The study was approved by the University Ethics Committee.

### Materials and Method

Children completed the 20 item Kiddie Mach (Christie & Geis, 1970) at the beginning of the school year, using a five point Likert scale (1 = *strongly disagree* to 5 = *strongly agree*). An example item is “It is never right to tell a lie”. Some items were reverse scored such that higher scores represent higher Machiavellianism. The scale demonstrated reliability of .58.

Cameras were placed unobtrusively at vantage points from which the playgrounds were visible and children were videotaped during recess. These vantage points meant that we could not record sound, but allowed a full view of the playground without the children knowing they were being filmed. Camera operators utilized a table of numbers that represented participant IDs, selected at random from all participants in the school. Children identified by the numbers were videoed on that day and video operators followed the child for as long as possible at that time. Videoing would stop for that child when he/she was no longer visible and videoing of the next child on the table of random numbers would begin.

Each target participant was observed in 39 recesses, which equated to one observation for each week of the school year; each period of observation lasted on average 18 minutes. If the child was away from school in any given week, an additional observation was collected the following week. Whilst we ensured that all data for a given child were collected within the same school year, data collection for the full sample took place over four years. No children joined or left the study. All observations of a target participant were coded in Observer XT 9 (Noldus, Netherlands) by coders who were blind to the Machiavellianism scores. Using Observation coding was undertaken by a total of sixteen trained undergraduate and postgraduate students and four members of staff who were required to reach an acceptable level of inter-rater reliability with practice videotapes (Intra-Class Correlations >.80) before they were able to code data. Assessments of reliability were conducted throughout the study to avoid observer drift (Pellegrini, 1996).

Within Observer XT, the data were coded across time using continuous event sampling. In the current study, data represent the percentage of observation time engaged in specific behaviour; for social monitoring, data represent the percentage of observed time in social groups when the child was seen to be observing peers outside of their immediate social group. Reliability between observers was assessed using Intra-Class Correlations (ICC) across 5% of the observations. Reliability was moderate to high for the observed variables in the current study and exact details are noted below for each behavioural code. A high number of interactions (95%) were same-gender as found in previous research (e.g., Blatchford, Baines, & Pellegrini, 2003). The following are the categories of behaviour observed.

#### Aggression

was categorised as direct or indirect aggression. Direct aggression was categorised as the target engaging in physical aggressive acts against another child (ICC = .81). Indirect aggression was categorised as the target deliberately engaged in ignoring another person(s) during active conversation or ostracizing them from interaction when engaged within the group (ICC = .76).

#### Peer acceptance

was assessed with target-initiated acceptance and other-initiated acceptance. Target-initiated acceptance was coded when the target child made a social overture that another child accepted (ICC = .84). Other-initiated acceptance was coded when another child who had been alone or in another group made a social overture that the target child responded positively to (ICC = .78). That social overture might have been a tap on the shoulder, speaking to the other person, or trying to get their attention another way (e.g. starts play).

#### Peer rejection

was measured by target-initiated rejection and other-initiated rejection. Target-initiated rejection was coded when the target child made a social overture that another child ignored (e.g. turning their back on the target child) (ICC = .92). Other-initiated rejection was coded when another child initiated interaction that was rejected by the target child, as demonstrated by the target child turning their back on them or walking away from them (ICC = .95).

#### Social monitoring

was categorized when the target participant was watching another person or group outside of their immediate social group (ICC = .81). Data represent percentage of time monitoring others when only engaged in social groups and not the total observed time.

## Results

The small number of participants does not permit examination of whether behaviour elicited in peer relationships changed over the school year or whether Machiavellianism scores predicted that change using latent growth curve modelling (LGCM) techniques. However, an analysis of behavioural change was conducted on the larger sample of participants (*N* = 149) observed as part of the Lancashire Longitudinal Study of Social and Emotional Development. Findings, using LGCM in Mplus (Muthén & Muthén, 1998-2007) showed behaviour were stable over time for boys and girls, with individual differences only in starting point (girls: CFI ≥ .943, TLI ≥ .946, SRMR ≥ .050; boys: CFI ≥ .948, TLI ≥ .951, SRMR ≥ .078). Variances showed that the intercepts for each behaviour were significant, (girls: β_0_ ≥ .314, *p* < .001; boys: β_0_ ≥ .343, *p* < .001), but the slopes were not (girls: β_1_ ≤ .027, *p* ≥ .183; boys: β_1_ ≤ .018 *p* ≥ .436). Thus, employing proportion of time for each behaviour across the full school year is appropriate because there were no significant changes in behaviour over the school year. Such findings have been demonstrated before (Blatchford et al., 2003).

Data for the children in the current study were skewed and non-normal. Transformations were conducted on the data, but these failed to produce an acceptable normal distribution. Therefore, we have conducted spearman’s rank correlations to account for this. Due to the skewed data and small sample size further analyses were not conducted.

### Behavioural Measure Correlations

For boys, positive relationships were identified between Machiavellianism and direct aggression, indirect aggression, target-initiated acceptance, and other-initiated acceptance. This suggests that for boys, higher levels of Machiavellianism are associated with an increased amount of time engaging in direct and indirect aggression, having their bid to join other groups accepted and accepting other children in to their group. Furthermore, the results show that Machiavellianism is negatively correlated with target-initiated rejection, suggesting that higher Machiavellianism scores are associated with boys spending less time rejecting their peer’s bids to join the group. For girls, Machiavellianism is negatively correlated with indirect aggression, target-initiated acceptance, other-initiated rejection, and other-initiated acceptance. Findings suggest that girls with higher Machiavellianism scores spend less time engaging in indirect aggression and less time having their bids to join other groups accepted. Girls with higher Machiavellianism scores spend less time accepting and rejecting their peer's attempts to join them in social interaction. Machiavellianism in boys and girls was not related to increased social monitoring of others outside of their social group. These correlations are shown in Table 1.

**Table 1:** Mean Proportion of Time Engaged in Each Behaviour and Correlations Between Machiavellianism and the Behavioural Measures for Boys and Girls

	*M*	*SD*	MA	DA	IDA	TIR	TIA	OIR	OIA	LOG
MA	50.00	8.26	–	.21	.27	-.21	.50*	.02	.26	-.07
DA	.82	1.22	-.12	–	.42	.26	.36	.29	.66**	.52*
IDA	.56	.90	-.47	.28	–	.19	.11	.00	.14	.05
TIR	.28	.32	-.03	-.03	.02	–	-.10	.52*	.21	-.42
TIA	2.63	1.70	-.46	.45	.44	-.28	–	.02	.41	.39
OIR	.92	1.28	-.40	.08	.19	-.06	.22	–	.33	-.29
OIA	2.75	3.90	-.41	.63**	.36	-.04	.41	.33	–	.39
LOG	56.27	12.59	-.16	.51*	.31	.16	.22	.06	.18	–

*Note.* Correlations for boys above the diagonal and correlations for girls below the diagonal. Looking outside of group is the proportion of time the child looked outside of the social group to observe another social group or individual.

MA: Machiavellianism; DA: Direct Aggression; IDA: Indirect Aggression; TIR: Target Initiated Rejection; TIA: Target Initiated Acceptance; OIR: Other Initiated Rejection; OIA: Other Initiated Acceptance; LOG: Looking Outside Group (social monitoring).

*p < .05. **p < .01.

## Discussion

The current study expands on previous Machiavellianism studies that have used self and other reports of behaviour by demonstrating that Machiavellianism is associated with children’s directly observed social interactions with peers. Boys with higher Machiavellianism scores engaged in more direct and indirect aggression, but also engaged in positive behaviour by accepting peers into their own social group. Girls with higher Machiavellianism scores spent less time engaging in social exclusion behaviour, being accepted by other children into other social groups and rejected and accepted peers less in to their own social group.

For boys, higher levels of Machiavellianism were associated with accepting their peer's bids to join their own social group, having their own overtures to join other groups accepted, increased aggression, and indirect aggression (social exclusion behaviour). Such behaviour may be evidence of using both prosocial and coercive strategies (Hawley, 2003). Accepting others into their group is a prosocial act, but aggressive behaviour towards their peers is evidence of coercive behaviour; engaging in both behaviour may increase their success at attaining resources and increase social dominance (Hawley, Little, & Card, 2008).

Girls with higher Machiavellianism displayed fewer social exclusion behavior (e.g. turning their back on their peer) and also spent less time getting accepted by their peers. Research typically reports girls’ greater engagement in indirect aggression (Archer, 2004) and more intimate peer networks (Rotenberg, MacDonald, & King, 2004), which may facilitate their greater willingness to engage in indirect aggression (Burr, Ostrov, Jansen, Cullerton-Sen, & Crick, 2005). We, therefore, expected Machiavellianism to increase girls’ use of indirect aggression, but this was not the case. Indirect aggression becomes more prevalent in girls around the age of 11 (Björkqvist, Österman, & Lagerspetz, 1994), and the young girls in the present study may not have the skills to employ social exclusion tactics. Furthermore, Machiavellianism in girls at this developmental stage may not be related to socially exclusive behaviour, but other forms of indirect aggression. Sutton and Keogh (2000) suggest Machiavellianism may be linked to indirect (bullying) behaviour such as gossip, and failure to find the predicted relationship between Machiavellianism and indirect aggression may reflect girls’ use of gossip, which could not be analysed in this study because we did not have sound to accompany our video footage. Investigating the use of gossip is also important to explore as it may explain the relationship between Machiavellianism and less time being accepted by others for girls high on Machiavellianism. Machiavellianism has been shown to be associated with prosocial and coercive strategies (Hawley, 2003), and these girls may employ coercive strategies to attempt to gain group acceptance. But, it is possible that other girls, even at this young age, see this behaviour as threatening, and, then, deny group acceptance. However, this is only speculation and future research should focus on the actual strategies employed by children high on Machiavellianism when attempting to join other children’s social groups.

In the current study we found that girls with higher levels of Machiavellianism spent less time accepting peers, but also less time rejecting peers into their own social group. This behaviour appears inconsistent and strongly suggests, first, that accepting and rejecting are not always reciprocal, supporting previous work (Sentse, Lindenberg, Omvlee, Ormel, & Veenstra, 2010). Machiavellianism in childhood is associated with inconsistent behaviour such as the use of prosocial and coercive strategies and being both liked and disliked by their peers. Using both prosocial and coercive strategies allow for adaptability when employing tactics to attain their goals which may also explain their controversial status among peers (Hawley, 2003; Palmen, 2009). However, the reasons that Machiavellian girls both reject and accept their peers less in to their own group remain unclear. Future research should investigate why children high on Machiavellianism choose to reject or accept other children’s overtures for social contact and in particular whether it is based on particular characteristics of the child making the bid to join or on specific dynamics of the current social situation. It appears to be the case that acceptance and rejection are more complex than initially thought and a more in depth observational study is warranted.

Machiavellianism is characterised by distrust of others (Christie & Geis, 1970) and associated with anxiety in children (Poderico, 1987). Thus, we expected to find a positive relationship between Machiavellianism and social monitoring because monitoring may inform decisions relating to whether an individual is a threat or can join their group. However, we did not find any relationships between Machiavellianism and social monitoring behaviour for boys or girls. It is possible that Machiavellian traits in childhood are unrelated to social monitoring, or children could learn strategies of social monitoring as they mature. Future research should examine the role of social monitoring at a later stage of development.

### Limitations

This is a preliminary study and limited by the small number of children participating. Future research should include larger samples across different age ranges, together with additional measures of personality and speech. Machiavellianism has been found to correlate with Narcissism and Callous-Unemotional traits (Kerig & Stellwagen, 2010) and future work should include these traits as potential controls. The analysis of social discourse will also provide a wealth of information relating to the verbal manipulation of peers and association with Machiavellianism, extending research suggesting Machiavellianism may be linked to behaviour such as gossip (Sutton & Keogh, 2000). We also note the low Cronbachs alpha for the Kiddie Mach measure. Although this is consistent with information about the scale reported by Christie and Geis (1970), this is lower than the relatively few other studies using the Kiddie Mach. Therefore, we suggest caution when interpreting the findings and recommend future observational research.

To conclude, the current research is a preliminary study that highlights the utility of the observational method for Machiavellianism research. We show that Machiavellianism is associated with children’s actual social behaviour with peers and that there are gender differences in how Machiavellianism affects social behaviour with peers at this stage in development. This is clearly highlighted by the unexpected gender differences for engaging in indirect aggression, with Machiavellian boys spending more time engaged in that behaviour. Future research should consider gender when investigating Machiavellianism and behaviour in childhood. Based on the findings from the current study, future research should attempt to establish a profile of Machiavellianism through the continued use of the observational method, but with a larger sample of males and females, observations at different stages of development, and the collection of social discourse data.
